# Screening and Treatment Intervention for Insulin Resistance/Hyperinsulinemia in the General Population to Prevent Heart Failure or Slow Its Worsening

**DOI:** 10.3390/biomedicines14071571

**Published:** 2026-07-14

**Authors:** Serafino Fazio, Flora Affuso, Valeria Fazio, Valentina Mercurio

**Affiliations:** 1 Department of Internal Medicine, Federico II University, 80131 Naples, Italy; 2 Independent Researcher, 80129 Naples, Italy; flora.affuso70@gmail.com; 3 Cardiologia, Ospedale Santa Maria delle Grazie, 80078 Pozzuoli, Italy; valeria.fazio1986@gmail.com; 4 Dipartimento di Scienze Mediche Traslazionali, Federico II University, 80131 Naples, Italy; valentina.mercurio@unina.it

Despite significant progress in the prevention and treatment of cardiovascular diseases in recent decades, they remain the leading causes of death worldwide [1]. In particular, heart failure, the common endpoint of most heart diseases, affects between 1 and 3% of the adult population worldwide, but increases to approximately 10% in adults over 70 years of age, and exceeds 20% in those over 80–85 years of age [2,3]. HF is the leading cause of hospital admissions in Italy, accounting for approximately 2% of total hospital admissions, with high rehospitalization rates and very high healthcare costs [3]. In recent years, cases of HFpEF have been increasing steadily, particularly in women, and have now exceeded 50% of HF cases. It is well known that HFrEF and HFpEF have completely different causes and pathophysiological mechanisms, and that their therapies differ accordingly. In fact, while HFrEF has well-defined treatments according to guidelines, HFpEF has no clearly defined therapies [4]. Important recognized causes of HF, particularly HFpEF, are cardiometabolic diseases. Among these, insulin resistance, with associated hyperinsulinemia, is very prevalent in the general population of developed and developing countries [5,6]. It is the principal cause of type 2 diabetes, preceding its development by many years—even 15 or more [7,8]. In the meantime, hyperinsulinemia produces significant damage to the cardiovascular system, to the extent that individuals with type 2 diabetes are treated, according to guidelines, as if they had already experienced a cardiovascular event [9]. It has been shown that hyperinsulinemia associated with insulin resistance is also linked to concentric left ventricular remodeling and diastolic dysfunction, main features of HFpEF [10,11,12,13].

Therefore, to reduce hospitalizations, deaths, and healthcare costs resulting from HF, we believe that it would be extremely important to implement a more thorough cardiovascular disease prevention plan, particularly by intervening early in the recognition and treatment of insulin resistance in the general population, and finding treatments that more effectively block or slow the worsening progression of HF.

## References

[1] World Health Organization (2025). Cardiovascular Diseases (CVDs). https://www.who.int/news-room/fact-sheets/detail/cardiovascular-diseases-(cvds).

[2] HFSA-Heart Failure Society of America 2025-Scientific Statement. HFStats 2025: Heart Failure Epidemilogy and Outcome Statistics. https://hfsa.org/hf-stats-2025-heart-failure-epidemiology-and-outcomes-statistics.

[3] Malfatto G., Soranna D., Mazzucco R., Villani A., Giglio A., Zambon A., Parati G. (2026). Prevalence and incidence of heart failure hospitalizations from 2005 to 2023 and determinants of optimal treatment implementation in clinical practice: A population-based study in Northern Italy. Int. J. Cardiol. Cardiovasc. Risk Prev..

[4] Borlaug B.A., Sharma K., Shah S.J., Ho J.E. (2023). Heart Failure with Preserved Ejection Fraction: JACC Scientific Statement. J. Am. Coll. Cardiol..

[5] Hill M.A., Yang Y., Zhang L., Sun Z., Jia G., Parrish A.R., Sowers J.R. (2021). Insulin resistance, cardiovascular stiffening and cardiovascular disease. Metabolism.

[6] Ormazabal V., Nair S., Elfeky O., Aguayo C., Salomon C., Zuñiga F.A. (2018). Association between insulin resistance and the development of cardiovascular disease. Cardiovasc. Diabetol..

[7] Accili D., Deng Z., Liu Q. (2025). Insulin resistance in type 2 diabetes mellitus. Nat. Rev. Endocrinol..

[8] Freeman A.M., Acevedo L.A., Pennings N. (2026). Insulin Resistance. StatPearls [Internet].

[9] Cosentino F., Grant P.J., Aboyans V., Bailey C.J., Ceriello A., Delgado V., Wheeler D.C. (2020). ESC Scientific Document Group. 2019 ESC Guidelines on diabetes, pre-diabetes, and cardiovascular diseases developed in collaboration with the EASD. Eur. Heart J..

[10] Sundström J., Lind L., Nyström N., Zethelius B., Andrén B., Hales C.N., Lithell H.O. (2000). Left ventricular concentric remodeling rather than left ventricular hypertrophy is related to the insulin resistance syndrome in elderly men. Circulation.

[11] Ho J.E., Chen Z.Z., Lau E.S. (2025). Insulin Resistance and Diabetes in HFpEF: Bystander or Instigator?. J. Am. Coll. Cardiol..

[12] Fazio S., Mercurio V., Fazio V., Ruvolo A., Effused F. (2024). Insulin Resistance/Hyperinsulinemia, Neglected Risk Factor for the Development and Worsening of Heart Failure with Preserved Ejection Fraction. Biomedicines.

[13] Gudenkauf B., Shaya G., Mukherjee M., Michos E.D., Madrazo J., Mathews L., Shah S.J., Sharma K., Hays A.G. (2024). Insulin resistance is associated with subclinical myocardial dysfunction and reduced functional capacity in heart failure with preserved ejection fraction. J. Cardiol..

